# A Discussion of Whether Various Lifestyle Changes can Alleviate the Symptoms of Irritable Bowel Syndrome

**DOI:** 10.3390/healthcare10102011

**Published:** 2022-10-12

**Authors:** Yohei Okawa

**Affiliations:** Department of Psychosomatic Medicine, Tohoku University Graduate School of Medicine, Sendai 980-8575, Japan; yohei-tky@umin.ac.jp; Tel.: +81-80-54833933

**Keywords:** functional gastrointestinal disorders, FGIDs, irritable bowel syndrome, IBS, defecation disorder

## Abstract

Irritable bowel syndrome (IBS) causes abdominal pain during bowel movements and is diagnosed according to the Rome IV international diagnostic criteria. Patients diagnosed as having IBS experience abdominal pain at least 1 day/week, on average, over a 3-month period and not 3 days per month. A diagnosis of IBS is confirmed if symptoms have persisted for more than 6 months. IBS symptoms negatively affect daily life. First, improving daily habits are important to ameliorating IBS symptoms. IBS symptoms can be alleviated by staying active, sleeping, resting and staying stress-free. In addition, it is important to eat three, balanced meals a day on a regular basis and avoid overeating, especially at night. Spicy foods, high-fat foods, and alcohol can exacerbate symptoms. Researchers found, in a literature review, that IBS symptoms can be ameliorated by improving daily habits, thus relieving abdominal pain and the defecation symptoms of IBS.

## 1. Introduction

Functional gastrointestinal disorders (FGIDs) are disorders characterized by chronic or recurrent gastrointestinal symptoms, the absence of organic lesions on laboratory examination, and symptom-induced dysfunction [1]. Types of FGIDs include irritable bowel syndrome (IBS), functional bloating, functional constipation, functional diarrhoea, and unspecified functional bowel disease [1,2,3]. The symptoms of IBS, a classic functional gastrointestinal disorder, include abdominal pain, discomfort, and associated bowel abnormalities [3]. The Rome IV diagnostic criteria are used to diagnose IBS. According to the Rome IV diagnostic criteria, IBS is characterized by abdominal pain related to defecation or defecation frequency occurring at least 1 day per week in the past 3 months. Two or more of the above three items related to changes in stool shape can be used to diagnose a patient with IBS in addition to symptoms that have persisted for 6 months [4].

Subtypes of IBS can be divided into IBS with diarrhoea (IBS-D), IBS with constipation (IBS-C), mixed IBS (IBS-M), and unclassifiable IBS (IBS-U). These subtypes are believed to be useful in clinical practice and therapy. The Rome IV criteria are only used to assess subjective symptoms [4]. Therefore, it is difficult to apply the Rome IV diagnostic criteria to patients who are unconscious or cognitively impaired [5]. Therefore, professional medical examination and clinical judgement may be needed. In addition, stool morphology in IBS patients varies from watery to hard stools [6], suggesting that gastrointestinal transit time is associated with stool morphology [7,8,9]. However, there are also reports that the frequency of defecation and gastrointestinal transit time in IBS patients are the same as those in healthy subjects [10,11]. Gastrointestinal transit time may require further scientific validation. Thus, IBS causes intestinal motility disorders, such as constipation and diarrhoea and abdominal pain associated with abnormal defecation, and the defecation symptoms differ for each IBS subtype. The four subtypes of IBS according to the Bristol stool form scale (BSFS) are IBS-C, IBS-D, IBS-M, and IBS-U, but depending on the subtype, IBS is also a risk factor for faecal incontinence and has been reported to reduce the quality of life (QOL) of patients [12,13,14]. All IBS subtypes adversely affect daily life, so it is important to first improve daily life habits to alleviate symptoms.

## 2. Materials and Methods

In this study, the authors suggest that some lifestyle changes can alleviate the defecation symptoms of irritable bowel syndrome. In this review, of current literature, the authors discuss existing knowledge, challenges and future prospects. PubMed was searched and yielded the cited references; the papers were published in English. Additionally, papers with scientific knowledge that were published in academic journals were retrieved. The search keywords for extracting these papers were “functional gastrointestinal disease, FGID”, “irritable bowel syndrome, IBS”, and “daily lifestyle habits”. This is a review of the literature regarding these subjects. There were no restrictions on the date of publication, sample size, research design or age of the subjects, and we only cited published papers with scientific knowledge and consensus.

## 3. Results and Discussion

### 3.1. Understanding the Factors Related to the Onset of IBS

**Figure 1:**
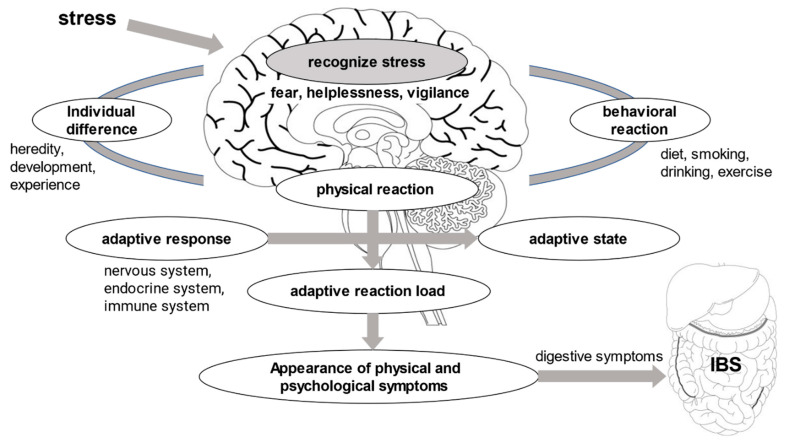
Factors related to IBS symptoms and adaptive responses.

Factors associated with the development of IBS can be related to genetics, environment, infection, inflammation, gut microbiota, or stress [3]. Stress, a psychosocial factor, plays a major role in the development of hyperalgesia and gastrointestinal motility disorders [15,16] (Figure 1). Additionally, poor daily life habits, such as poor diet, lack of exercise, and inadequate sleep, can cause symptoms in IBS patients [17]. These factors can have a significant negative impact on daily life, thus reducing the patient’s quality of life. Additionally, eating habits are important in typical lifestyle habits. Food intake is an essential part of everyday life. However, dietary intake may affect gastrointestinal transit time and alter defecation symptoms when certain foods are ingested [9,18]. Therefore, it is important to examine how specific types of foods affect bowel symptoms.

In our daily lives, people are subject to experience various stresses related to home life, work environment, school, neighbours, and so on. The susceptibility to this stress varies greatly depending on the individual’s genetic and environmental factors, and the method and degree of adaptation also vary greatly among individuals. If the stress is within the tolerance of the adaptive response, no major problems will emerge, but when the stress exceeds the individual’s ability to adapt, various physical and mental disorders can occur. One of these diseases is IBS.

### 3.2. Improving Daily Habits

In everyday life, people are exposed to a wide variety of stresses related to home life and work environment. Susceptibility to these stresses varies greatly depending on the individual’s genetic and environmental factors. In addition, the method and degree of adaptation also vary greatly from person to person. If the stress is within the tolerance of the adaptive response, there is no problem, but if the magnitude of the stress exceeds the individual’s ability to adapt, various physical and mental disorders may appear [15]. Various daily lifestyle habits, such as exercise, sleep, and other behaviours (smoking, drinking), are important in IBS (Table 1). In a study on exercise therapy, researchers verified the effectiveness of moderate exercise under the guidance of professional staff in approximately 100 IBS patients. Although a high dropout rate was observed among the participants in the study, IBS symptoms were significantly alleviated in the moderate exercise group [15,19].

In a systematic review of exercise therapy, researchers reported that exercises such as yoga, walking and aerobics were effective treatments for IBS [20]. Therefore, exercise under appropriate guidance is thought to ameliorate IBS symptoms.

In studies on the relationship between IBS and sleep conducted in other countries, researchers reported that IBS was significantly associated with sleep disturbances [21,22]. Additionally, in Japan, it has been reported that the frequency of IBS was higher in the group in which sleep induction was difficult [23]. However, there is currently no clear evidence that reducing the incidence of sleep disturbances mitigate IBS symptoms.

Studies on alcohol consumption have shown that high alcohol consumption over a short period of time exacerbates diarrhoea symptoms, but light to moderate consumption did not result in a significant difference in the incidence of exacerbated IBS symptoms [24,25]. Therefore, there is no clear evidence that changing alcohol consumption habits alleviates IBS symptoms. There was also no significant association observed between smoking and IBS symptoms [22].

Moreover, exercise therapy is effective at reducing IBS symptoms, and implementation under appropriate guidance is expected. However, there is no evidence that IBS symptoms can be ameliorated by improving sleep habits and avoiding other behaviours (smoking and drinking) [1,3,15].

### 3.3. Improving Eating Habits, Which Are Essential in Daily Life

There are various daily lifestyle habits, such as exercise, sleep, and other behaviours, but “diet intake” is mentioned as a lifestyle habit that is particularly related to IBS symptoms (Table 1). General dietary advice to alleviate IBS symptoms includes eating regular meals and drinking plenty of fluids. It has been reported that diets that are likely to cause IBS symptoms involve foods containing many lipids, caffeine, and spices [15,24,26]. In some studies, it has been reported that a high-fat diet exacerbates IBS symptoms [27]. In addition, lipid infusion into the duodenum has been shown to exacerbate gastrointestinal symptoms [28]. Caffeine stimulates rectosigmoid motility of the large intestine and exacerbates IBS symptoms [29]. Regarding spices, it has been suggested that there is a relationship between cooking with red chilies and IBS symptoms. Capsaicin, the main ingredient in red chili, increases gastrointestinal motility and may cause abdominal burning and pain [30]. It has been reported that the frequency of eating foods such as curry and those rich in pepper, ginger, cinnamon, turmeric, etc., is significantly associated with an increased incidence of IBS [26,31]. The elimination of milk and dairy products has been reported to significantly alleviate IBS symptoms in lactose-intolerant IBS patients who followed a lactose-restricted diet for 6 weeks [32]. Hence, it is suggested that IBS symptoms are alleviated by refraining from eating foods that tend to induce IBS symptoms in daily life. Such food restrictions can be implemented in all aspects of daily life, including at home, work and school, and are very feasible.

In addition, an antigen-free diet based on IgG antibodies has been reported to reduce the occurrence of IBS symptoms and improve quality of life [33]. In a study in which a group of IBS patients who ate a diet in which all foods that increased the concentration of IgG antibodies were eliminated (elimination diet group) was compared with a control group, the IgG antibody-free diet group exhibited significantly less IBS symptoms [33].

In addition, studies in other countries have shown that avoiding a diet rich in short-chain carbohydrates, including “fermentable” carbohydrates, “oligosaccharides”, “disaccharides”, “monosaccharides” and “polyols”, alleviated IBS symptoms [26,34,35]. These ingredients are known as “FODMAP” by their acronym. Typical FODMAP-rich foods include wheat, onions, chickpeas, lentils, apples, corn, milk, yogurt, and honey [26,34,36,37,38]. Foods rich in these FODMAPs are difficult to decompose and absorb in the small intestine and continue to the large intestine without processing, where they are rapidly fermented and decomposed by intestinal bacteria. During the process, hydrogen gas and methane gas are generated, and they draw water into the intestinal tract due to their high osmotic pressure. In clinical trials, it has been reported that IBS patients who ate a low-FODMAP diet had fewer IBS symptoms than those who ate a normal diet [34,35,39,40,41,42,43].

Furthermore, it has been reported that psyllium, a dietary fibre, is effective in alleviating IBS symptoms [36,37,38,44,45,46,47]. There are two types of dietary fibre: soluble fibre (such as psyllium and ispaghula) and insoluble fibre (wheat bran). Soluble fibre forms a gel in the intestinal tract, thus facilitating the movement of stool masses. Insoluble fibre, however, absorbs water into its fibrous structure and swells, increasing the volume of the stool itself and stimulating intestinal peristalsis. Psyllium, a soluble fibre, is effective in ameliorating the common symptoms of IBS, as well as IBS-related constipation symptoms [37]. However, wheat bran, an insoluble fibre, has been reported to be effective for IBS-related constipation symptoms, but worsens general IBS symptoms and IBS-related abdominal pain [36]. In a recent systematic review, although researchers confirmed the efficacy of consuming soluble fibre for alleviating symptoms in IBS patients, the use of insoluble fibre was not confirmed and data on dietary fibre intake in IBS patients are limited [38,44,45].

Moreover, although improvements in dietary habits are effective in IBS patients, there are also challenges. Dietary guidance for IBS patients may need to be divided into two categories: useful items that can be suggested to all patients; and individualized guidance. All patients are advised to refrain from snacking and skipping meals and to maintain regular eating habits. In addition, recognizing and restricting foods and dietary components that contribute to the manifestation of IBS symptoms should be considered on a patient-by-patient basis.

### 3.4. Improving Bowel Habits in Daily Life

Regular review of bowel habits may reduce the occurrence of irregular bowel movements due to IBS. If rectal sensation is normal, it is recommended that patients defecate as soon as possible without resistance. However, it has been reported that when rectal sensation is reduced, the symptoms of faecal incontinence can be significantly alleviated if patients attempt to defecate even when there is no urge [46,47]. Nevertheless, in patients with spinal cord disorders and in elderly people, rectal sensation is reduced, so even if there is a faecal mass in the rectum, faeces may continue to accumulate in the rectum without the individual feeling the urge to defecate. In that case, overflow incontinence or leaky faecal incontinence may occur, so it is recommended that these individuals attempt to defecate twice a day (approximately 30 min after breakfast or dinner), even when there is no urge (stress defecation may help) [46,48]. Nurse-initiated education and advice on defecation also reduces the incidence of symptoms related to faecal incontinence and benefits caregivers [47]. In any case, it is important to review and understand defecation habits related to IBS symptoms.

## 4. Conclusions

Functional gastrointestinal disorders (FGIDs) are associated with chronic or persistent gastrointestinal symptoms. Laboratory examination often reveals no organic lesions, and symptoms are due to functional impairment. One of the most common FGIDs is irritable bowel syndrome (IBS). In patients with IBS, bowel movements associated with abdominal pain occur, and the Rome IV diagnostic criteria, which are international diagnostic criteria, are used for its diagnosis. The diagnostic criteria include abdominal pain that recurs on average at least 1 day/week for 3 consecutive months and not 3 days per month, and a change in stool shape. An IBS diagnosis is confirmed if the symptoms have persisted for more than 6 months. IBS is further divided into four subtypes based on the frequency of bowel movements, according to the Bristol stool form scale (BSFS). The subgroups of IBS are IBS with constipation type (IBS-C), IBS with diarrhoea type (IBS-D), IBS with mixed type (IBS-M), and IBS with unclassifiable type (IBS-U). IBS symptoms negatively affect daily life. To relieve IBS symptoms, it is important to first teach patients about their daily lifestyle habits so that they may improve them.

First, as a premise, guidance for alleviating IBS symptoms is difficult to generalize because there are substantial individual differences in lifestyle habits. In Japan, people normally eat three meals a day, but the food cultures of other countries are different. In general, guidance on lifestyle habits for relieving IBS symptoms is limited. However, in research reports from other countries, researchers have confirmed the effectiveness of a low-FODMAP diet and soluble dietary fibre [9,10,11,12]. In Japan, a large-scale randomized controlled trial has not been sufficiently conducted, and future research is expected. Therefore, the authors propose the following.

First, IBS symptoms may be alleviated by reducing stress, staying active, sleeping and resting. In addition, it is important to eat three, balanced meals on a daily basis, and avoid overeating, especially at night. Spicy foods, high-fat foods, and alcohol can exacerbate symptoms. Thus, based on the literature review, researchers suggest that IBS symptoms can be relieved by improving daily habits, thus alleviating abdominal pain and defecation symptoms due to IBS.

## Figures and Tables

**Table 1:** Daily lifestyle guidance to improve IBS symptoms.

Guidance for Daily Life	Reconsidering One’s Daily Habits and Removing Factors That Exacerbate Symptoms.
	It is important to maintain a stable lifestyle rhythm by paying attention to regular and correct meals, bowel movements, and sleep.
	Stress management relieves the feeling of stress by changing stressful moods (light exercise, hobbies, etc.) during high stress.
**Guidance for dietary**	IBS patients with diarrhoea should be careful about ingesting foods that stimulate the intestinal tract and cause IBS symptoms (caffeine, alcohol, soft drinks, etc.).
	Typical foods that exacerbate IBS symptoms include short-chain carbohydrates (FODMAP), high-fat foods, and spices.
	There are various reports on the effect of dietary fibre on IBS symptoms, but intake of soluble dietary fibre improves bowel movements.
**Guidance to improve stress sensitivity**	Since there are individual differences in how stress is felt, even in the same environment, the way people perceive it differs greatly. Psychotherapy, such as cognitive behavioural therapy, is performed to improve sensitivity to stress.
	IBS symptoms are closely related to everyday life. It is important to remove the factors that exacerbate the symptoms and maintain regular daily habits.

## Data Availability

Not applicable.
